# An early predictive model for acute respiratory distress-syndrome related to pancreatitis in pregnancy: an 8-year multicenter analysis

**DOI:** 10.1186/s40001-025-03223-w

**Published:** 2025-10-07

**Authors:** Yu Wang, Shu Zhou, JinJin Zhang, LiuRong Zhang, Qi Yang, YiDi He, GengYun Sun

**Affiliations:** 1 Department of Emergency Medicine, The First Affiliated Hospital of Anhui Medical University, Hefei City, Anhui Province China; 2 Department of Emergency Medicine, The Second Affiliated Hospital of Anhui Medical University, Hefei City, Anhui Province China; 3 Department of Epidemiology and Health Statistics, Anhui Medical University, Hefei City, Anhui Province China; 4 Department of Clinical Medicine, Anhui Medical University, Hefei City, Anhui Province China; 5 Department of Respiratory and Critical Care Medicine, The First Affiliated Hospital of Anhui Medical University, Hefei City, Anhui Province China

**Keywords:** Acute respiratory distress-syndrome, Acute pancreatitis in pregnancy, Predictive model

## Abstract

**Background:**

Acute respiratory distress syndrome (ARDS) related to acute pancreatitis in pregnancy (APIP) is associated with a higher risk of maternal and fetal death. This study aimed to explore early predictors and develop a predictive model for ARDS associated with APIP aligned with the updated global ARDS definition.

**Methods:**

The APIP data of two hospitals over an 8-year period were retrospectively collected. The variables were analyzed using Least Absolute Shrinkage and Selection Operator regression, and binary logistic regression to build a predictive model, visualized with a nomogram. The performance of the predictive model was then evaluated.

**Results:**

Of 6597 patients with acute pancreatitis, 103 pregnant patients were included, and 24 pregnant patients had ARDS. Lower oxygen saturation as measured by pulse oximetry to fraction of inspired oxygen (SpO_2_/FiO_2_) ratio, elevated heart rate (HR), and total cholesterol (TCH) were identified as independent risk factors for ARDS in APIP. Compared to previous scoring systems, the predictive model was more discriminating between APIP and ARDS, with an area under the receiver operating characteristic curve of 0.926 (95% CI 0.864–0.988). Notably, the new model performed best when the prediction cutoff was set at 0.205 (sensitivity = 0.823, specificity = 0.958). Calibration and decision curve analyses confirmed the strong clinical utility and accurate risk prediction.

**Conclusion:**

A new accurate utility predictive model for ARDS related to APIP, including three simple variables (HR, TCH, and SpO_2_/FiO_2_), was constructed at admission. Elevated total cholesterol level was first identified as an independent risk factor for ARDS in patients with APIP.

**Supplementary Information:**

The online version contains supplementary material available at 10.1186/s40001-025-03223-w.

## Introduction

Acute pancreatitis in pregnancy (APIP) is a rare but highly life-threatening gestational disease that may result in pancreatic necrosis, abscesses, and multiple organ dysfunction. Pancreatitis-associated acute respiratory distress syndrome (ARDS) is the earliest organ dysfunction in severe acute pancreatitis(SAP). The leading role of pancreatitis-associated ARDS in the development and high mortality rate of multiple organ dysfunction syndrome has been confirmed in SAP [1]. ARDS death toll accounts for 37.62% of SAP [2]. Pregnancy complicates this condition further. The fetal mortality rate was reported to be approximately 11.6–31.1% in APIP [3, 4], and pre-delivery ARDS was found to be an independent risk factor for fetal intrauterine death [4].

However, therapeutic options for APIP and ARDS are challenging. Early and rapid recognition of ARDS related to acute pancreatitis(AP), along with the prompt application of ventilatory support techniques, is crucial for improving outcomes [1]. Although various cytokines and inflammatory mediators such as interleukin-6 (IL-6), IL-8, protein C, angiopoietin-2, and miRNAs have been identified as predictors of ARDS, either individually or in combination [5, 6], these biomarkers are not obtained through routine examinations. Current scoring systems such as Acute Physiology and Chronic Health Evaluation II(APACHE II), Bedside Index for Severity in Acute Pancreatitis(BISAP), Systemic Inflammatory Response Syndrome(SIRS), Modified Marshall, and Ranson are used to assess the severity of AP in the general population, but specific time constraints, many variables, and complex processes may limit its practical application worldwide [7]. The physiology of pregnancy in AP is different from that of general patients with AP. These scores may not be applicable to the APIP. The lung injury prediction score (LIPS) has been used to evaluate the risk of ARDS in patients without AP [8] but has not been validated in APIP. Therefore, novel and uncomplicated predictors of ARDS related to APIP are required to complement these scoring systems.

Previous studies have reported prediction models for ARDS in patients with SAP based on the Berlin definition of ARDS [9–11]. However, these studies have rarely addressed unique physiological changes and considerations pertinent to pregnant women. As such, the existing models may not be directly applicable to this demographic. In addition, a new global definition of ARDS was built in 2023, which expanded the Berlin definition [12, 13]. Currently, very few studies have reported the predictive indicators of ARDS related to APIP based on the new definition. Given the physiological and anatomical changes during pregnancy and the potential harm of chest imaging radiation to the fetus, there is an urgent need for a predictive tool tailored for ARDS in the early phase of APIP.

This study aimed to identify early predictors and develop a straightforward predictive model for APIP-associated ARDS, aligning with the updated global ARDS definition. This model is intended for broad applications, particularly in emergency departments and settings with limited resources. Its implementation will enable medical professionals to promptly initiate proactive monitoring and treatment, and be practical for communication with patients and caregivers, thereby improving maternal and fetal outcomes.

## Methods

### Study design and patient selection

This study retrospectively analyzed the clinical data of patients with AP from the First Affiliated Hospital of Anhui Medical University and the Second Affiliated Hospital of Anhui Medical University in Hefei, China, from January 2016 to December 2023. Patients with APIP were included in the study. The inclusion criterion was acute pancreatitis diagnosed during pregnancy. Male patients with AP, female patients with AP with no pregnancy, and patients with APIP with insufficient medical data records were excluded. The study was conducted in accordance with the Declaration of Helsinki. The Ethics Committees of the First and the Second Affiliated Hospital of Anhui Medical University reviewed and approved this study(reference number PJ 2024–07-67,YX2024-181). Since the data used were anonymous and did not involve direct contact with participants, the Ethics Committees of the First and Second Affiliated Hospitals of Anhui Medical University waived the requirement for informed consent.

### Diagnosis and definitions

According to the 2012 revised Atlanta Criteria for AP [14], the diagnosis of acute pancreatitis requires two of the following three features: abdominal pain consistent with acute pancreatitis (acute onset of a persistent, severe, epigastric pain often radiating to the back); serum lipase level or serum amylase level (at least three times greater than the upper limit of the normal), and findings consistent with acute pancreatitis on imaging (contrast-enhanced CT[CECT], MRI, or abdominal ultrasound).

ARDS was diagnosed based on the 2023 New Global Definition of Acute Respiratory Distress Syndrome [12].

The new global definition of ARDS expanded the Berlin definition with the following recommendation [13]:

1. includes high-flow nasal oxygen at a minimum flow rate of ≥ 30 L/min.
2. used PaO_2_:FiO_2_ ≤ 300 mmHg or SpO_2_:FiO_2_ ≤ 315 (if SpO_2_ was ≤ 97%) to identify hypoxemia.
3. retains bilateral opacities for imaging criteria but adds ultrasound as an imaging modality, especially in resource-limited areas.

4 In resource-limited settings, positive end-expiratory pressure, oxygen flow rate, or specific respiratory support devices are not required.

### Data collection

Demographic information, Body Mass Index (BMI), history of gestational diabetes, gestational hypertension, and vital signs were collected from medical records at admission. Initial routine laboratory tests and arterial blood gas analysis were performed 24 and 48 h after admission. Complete blood count was examined using an automatic analyzer (SySMex XN9000, Sysmex Corporation, China). Biochemistry, including blood glucose, lipids, and electrolytes, was performed using an automatic biochemical analyzer (Cobas 8000, Roche Limited, Switzerland). Arterial blood gas analysis was performed using an automatic analyzer (i-STAT 300-G; Abbott Limited, USA).BISAP, APACHE II, and SIRS scores were evaluated in the first 24 h, and Modified Marshall and Ranson scores were calculated 48 h after admission.Chest CT and chest X-ray examinations were conducted on pregnant women only when severe pulmonary complications (such as respiratory failure, ARDS, or severe pneumonia) occurred and there was an urgent clinical need for imaging evidence to guide treatment, following full informed consent and the implementation of abdominal protective measures.

### Statistical analysis

Little's test for Missing Completely at Random (MCAR) was used to assess the nature of the missing data. To address the missing data issue, the Multiple Imputation (MI) method (predictive mean matching) was implemented to construct a complete database.

Data normality of the continuous variables was tested using the Shapiro–Wilk test. Normally distributed continuous variables are expressed as mean ± standard deviation and compared using a two-sided Student’s t-test. Non-normally distributed continuous variables were reported as median (interquartile range [IQR]) and were compared using the Mann–Whitney U test. Categorical variables were assessed as frequencies and percentages and compared using the chi-squared test or Fisher’s exact test. Continuous variables were analyzed in their original form to retain information. Least Absolute Shrinkage and Selection Operator (LASSO) regression were used to select predictor variables. The Variance Inflation Factor (VIF) and Tolerance were adopted to assess the issue of multicollinearity. Binary logistic regression analysis was used to further develop the predictive model, and a nomogram was formulated based on binary logistic regression.

The receiver operating characteristic (ROC) curve and area under the ROC curve (AUC) were used to evaluate the discriminative power of the predictive model,and an AUC > 0.7 indicated good model discrimination. The DeLong test was used to evaluate the difference in the AUC between the nomogram and scoring systems. Analyzing the gray zone based on model-predicted probabilities to optimize clinical decision-making. The gray zone is defined as the probability range with high uncertainty in model predictions. Calibration curves were plotted to measure the accuracy of the predictive models, reflecting the consistency between the predictions from the model and observed outcomes. A well-calibrated model indicates that the predictions lie on or near the 45° line in the calibration plot. The calibration curves were quantified using the Hosmer (H–L) goodness-of-fit test. The p-values were determined by the H–L test; *p* > 0.05, indicating that the model predictions were in best agreement with the standard diagnostic criteria. Decision curve analysis (DCA) was used to assess the clinical utility of the model, which showed the relationship between the probability thresholds for model predictions and the relative value of net benefit. By comparing the predictive mean matching (original analysis) and random forest (sensitivity analysis) imputation methods, the impact of imputation strategies on model performance was evaluated.

All statistical analyses and graphs were generated using R 4.2.3 software (R Foundation for Statistical Computing, Vienna, Austria), using the “mice,” “glmnet,” “autoReg,” “pROC,” “cutoff,” “fbroc,” “reportROC,” “rms,” “Resource Selection,” “ggDCA,” “rmda” and “ggplot2” packages. Differences with *p* < 0.05 were considered statistically significant.

## Results

### Baseline characteristics

In this study, out of 6597 patients with AP admitted to two hospitals from January 2016 to December 2023, 103 patients with APIP were finally included, with an average age of 29.53 ± 5.04 years old. Among them, 24 patients(23.30%) were diagnosed with ARDS and 79 patients(76.70%) were diagnosed without ARDS. During this period, 82539 pregnant women were admitted to the same hospitals and APIP occurred in 105 women.The incidence of APIP was 12.7 cases per 10,000 pregnant women (105/82539).

**Fig. 1:**
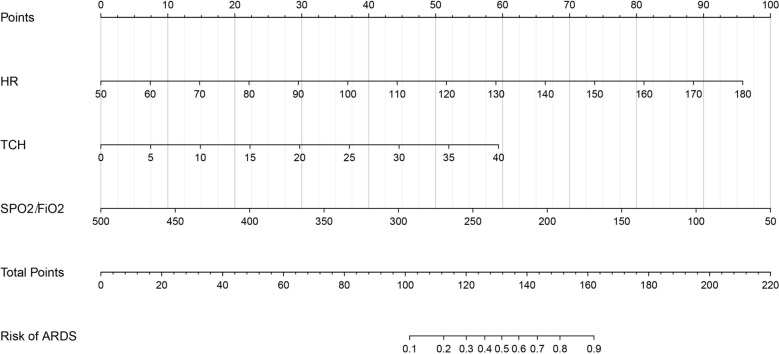
Nomogram of acute respiratory distress syndrome prediction model

Demographics, medical history, vital signs, arterial blood gas analysis results, laboratory findings, scoring systems at admission, and clinical outcomes were compared between patients complicated with and without ARDS. There were statistically significant differences in gestational weeks, trimester of pregnancy, etiology, occurrence time of AP, vital signs, scoring systems, and laboratory results of NLR, ALB, BUN, Calcium, CRP, PCT and total cholesterol(TCH) in the group of APIP patients with ARDS compared to the group of APIP patients without ARDS(*p* < 0.001 for all)(Table 1).

**Table 1:** Baseline characteristics of APIP patients complicated with or without ARDS

Characteristic	Patients without ARDS (n = 79)	Patients with ARDS (n = 24)	Total (n = 103)	*p* value
Demographic data				
Age	29.89 ± 4.79	28.38 ± 5.74	29.53 ± 5.04	0.200
BMI	23.51(26.67, 29.60)	27.34(26.67, 29.39)	27.00(23.94, 29.50)	0.229
Gestation weeks	28.43(32.86, 35.57)	34.21(31.04, 37.79)	33.86(29.43, 36.42)	0.046
Trimester of pregnancy, n(%)				0.007
The first trimester	4(5.10)	0(0.00)	4(3.90)	
The second trimester	13(16.50)	0(0.00)	13(12.60)	
The third trimester	62(78.40)	24(100.00)	86(83.50)	
Etiology, n(%)				0.004
Biliary	28(35.40)	3(12.50)	31(30.10)	
Hypertriglyceridemia	33(41.80)	19(79.20)	52(50.50)	
Others	18(22.80)	2(8.30)	20(19.40)	
Gestational hypertension, n(%)				0.421
No	75(94.90)	21(87.50)	96(93.20)	
Yes	4(5.10)	3(12.50)	7(6.80)	
Gestational diabetes, n(%)				0.365
No	60(75.90)	16(66.70)	76(73.80)	
Yes	19(24.10)	8(33.30)	27(26.20)	
Gravidity	2.00(1.00, 3.00)	2.00(1.00, 2.75)	2.00(1.00, 3.00)	0.585
Time of AP occurrence	1.00(1.00, 2.00)	1.00(0.50, 1.00)	1.00(1.00, 2.00)	< 0.001
Vital signs				
Temperature (℃)	37.00(36.50,37.50)	38.15(37.13, 38.58)	37.10(36.60, 37.80)	< 0.001
HR(bpm)	105.59 ± 19.62	129.46 ± 19.02	111.16 ± 21.88	< 0.001
RR(bpm)	20.00(20.00, 23.00)	25.00(21.25, 32.50)	21.00(20.00, 25.00)	< 0.001
MAP (mmHg)	87.00(80.30, 94.30)	94.00(88.50, 107.00)	88.60(81.60, 97.00)	0.002
SpO_2_/FiO_2_	338.00(303.00, 467.00)	236.50(194.00, 288.00)	334.00(270.00, 462.00)	< 0.001
Laboratory tests				
WBC (*10^9)	13.21(10.04, 16.12)	13.92(11.28,18.31)	13.23(10.16,16.70)	0.355
NLR	10.57(7.35, 15.42)	15.72(8.95,18.75)	11.80(7.38,17.38)	0.033
PLR	208.16(150.48, 253.54)	217.09(153.45,415.56)	208.16(151.52,268.83)	0.391
Hct (%)	34.47 ± 4.24	34.60 ± 5.09	34.50 ± 4.42	0.897
ALB (g/L)	32.80(28.10, 37.30)	25.20(21.58,30.40)	31.00(26.40,36.40)	< 0.001
TBIL (umol/L)	14.50(9.10, 24.70)	12.55(11.48,21.00)	14.10(10.20,23.10)	0.858
Scr (umol/L)	45.00(36.00, 53.90)	50.85(41.25,64.30)	46.80(36.30,55.00)	0.083
BUN (mmol/L)	2.70(1.94, 4.07)	3.87(2.26,5.27)	3.10(2.00,4.20)	0.099
Calcium (mmol/L)	2.12(1.94, 2.23)	1.80(1.33,2.18)	2.07(1.75,2.21)	0.012
CRP (mg/L)	27.31(5.66, 130.20)	157.20(96.73,205.05)	63.55(9.08,183.60)	< 0.001
PCT (ng/L)	0.12(0.05, 0.53)	0.49(0.18,1.02)	0.18(0.06,0.75)	0.001
TCH (mmol/L)	6.81(5.25, 9.90)	13.95(8.40,26.43)	7.59(5.27,13.55)	0.001
TG (mmol/L)	4.41(2.60, 14.90)	17.60(5.10,54.04)	5.93(2.75,19.56)	0.001
Glucose (mmol/L)	6.07(4.83, 8.29)	8.03(5.80,12.04)	6.49(4.91,8.71)	0.004
LDH (u/L)	199.00(162.00, 339.00)	278.00(229.00,573.25)	235.00(171.00,353.00)	0.005
PH	7.39(7.36, 7.43)	7.36(7.27,7.41)	7.39(7.33,7.43)	0.035
HCO_3_ (mmol/L)	17.90(13.90, 20.50)	14.75(10.55,16.78)	17.00(12.70,20.00)	0.005
Lac (mmol/L)	0.89(0.16, 1.27)	1.54(0.61,3.20)	1.02(0.21,1.48)	< 0.001
Scores				
24-h APACHE.II	4.00(2.00, 7.00)	12.00(10.00,16.75)	6.00(3.00,12.00)	< 0.001
48-h Modified Marshall	0.00(0.00, 1.00)	2.00(2.00,3.00)	0.00(0.00,2.00)	< 0.001
48-h Ranson	0.00(1.00, 2.00)	3.00(2.00,4.00)	1.00(1.00,3.00)	< 0.001
24-h BISAP	0.00(0.00, 2.00)	2.00(2.00,2.00)	1.00(0.00,2.00)	< 0.001
24-h SIRS	4.00(2.00, 5.00)	6.00(5.25,8.00)	4.00(3.00,6.00)	< 0.001
Outcomes				
Hospital stays (d)	9.00(6.00, 13.00)	17.00(13.00, 22.00)	11.00(7.00, 16.00)	< 0.001
ICU stays (d)	0.00(0.00, 1.00)	4.25(6.00, 8.75)	0.00(0.00, 4.00)	< 0.001
Invasive Ventilation, n(%)				< 0.001
No	75(94.90)	3(12.50)	78(75.70)	
Yes	4(5.10)	21(87.50)	25(24.30)	
Cesarean section, n(%)				0.001
No	35(44.30)	2(8.30)	37(35.90)	
Yes	44(55.70)	22(91.70)	66(64.10)	
Fetal outcomes, n(%)				< 0.001
Continue pregnancy	34(43.00)	0(0.00)	34(33.00)	
Fetal loss	1(1.30)	2(8.40)	3(2.90)	
Full-time birth	11(13.90)	8(33.30)	19(18.40)	
Prematurity	33(41.80)	14(58.30)	47(45.60)	
Newborn Birthweight (g)	2624.49 ± 656.43	2476.25 ± 860.36	2589.95 ± 707.38	0.442
0 min Apgar score	9.00(7.00, 10.00)	7.00(7.00, 9.00)	9.00(7.00, 10.00)	0.024
5 min Apgar score	10.00(9.00, 10.00)	9.50(9.00, 10.00)	10.00(9.00, 10.00)	0.054

*p* value < 0.05 was considered statistically significant

*ARDS* acute respiratory distress syndrome, *APIP* acute pancreatitis in pregnancy, *BMI* body mass index, *HR* heart rate, *RR* respiratory rate, *MAP* mean arterial pressure, *NLR* neutrophil-to-lymphocyte ratio, *PLR* platelet-to-lymphocyte ratio, *Hct* haematocrit, *Scr* serum creatinine, *TBIL* total bilirubin, *ALB* albumin, *Scr* serum creatinine, *BUN* blood urea nitrogen, *CRP* C-reactive protein, *PCT* procalcitonin, *TCH* total cholesterol, *TG* triglyceride, *LDH* Lactate dehydrogenase, *Lac* lactate, *SpO*_*2*_ oxygen saturation as measured by pulse oximetry, *FiO*_*2*_ fraction of inspired oxygen, *APACHE.II* acute physiology and chronic health evaluation II, Modified Marshall, modified Marshall scoring system, *BISAP* bedside index of severity in acute pancreatitis, *SIRS* systemic inflammatory response syndrome

### Clinical outcomes

APIP patients with ARDS had a greater likelihood of receiving invasive mechanical ventilation, cesarean section, longer length of ICU stay, and longer hospital stay(*p* < 0.001 for all). Fetal outcomes and 0 min Apgar score of newborns also showed significant differences between APIP patients with and without ARDS(*p* < 0.05 for both). Details are presented in Table 1.

### Predictors and model construction

Four variables (Temperature, heart rate(HR), TCH, SpO_2_/FiO_2_) were extracted using LASSO regression as predictors of APIP in patients with ARDS (E_Figure 1 and E_Table 1).The VIF values for the four variables are below 10, and the tolerance values are all above 0.1, indicating that there is no significant multicollinearity among the variables. (E_Table 2.) Further binary logistic regression analysis showed that a lower SpO_2_/FiO_2_ ratio (odds ratio: 0.98; 95%CI 0.97—0.99), elevated HR (odds ratio: 1.05; 95%CI 1.00—1.10), and elevated TCH (odds ratio: 1.11; 95%CI 1.02—1.20) were independent risk factors for ARDS related to APIP. In contrast, the temperature did not demonstrate significant independent predictive capability (*p* > 0.05)(Table 2).The above independent predictors were combined to construct a nomogram for the ARDS prediction model, excluding temperature (Fig. 1).

**Table 2:** Logistic Regression Model for predicting ARDS related to APIP

Characteristic	Univariate			Multivariate		
	Odds ratio	95%CI	*p* value	Odds ratio	95%CI	*p* value
Temperature	4.42	2.21–8.85	< 0.001	1.35	0.51–3.52	0.544
HR	1.07	1.03–1.10	< 0.001	1.05	1.00–1.10	0.050
TCH	1.14	1.07–1.22	< 0.001	1.11	1.02–1.20	0.013
SpO_2_/FiO_2_	0.98	0.97–0.99	< 0.001	0.98	0.97–0.99	0.004

*p* value < 0.05 was considered statistically significant

*ARDS* acute respiratory distress syndrome, *APIP* acute pancreatitis in pregnancy, *AUC* area under the curve, *CI* confidence interval, *HR* heart rate, *TCH* Total cholesterol, *SpO*_*2*_ oxygen saturation as measured by pulse oximetry, *FiO*_*2*_ fraction of inspired oxygen

### Model performance

**Fig. 2:**
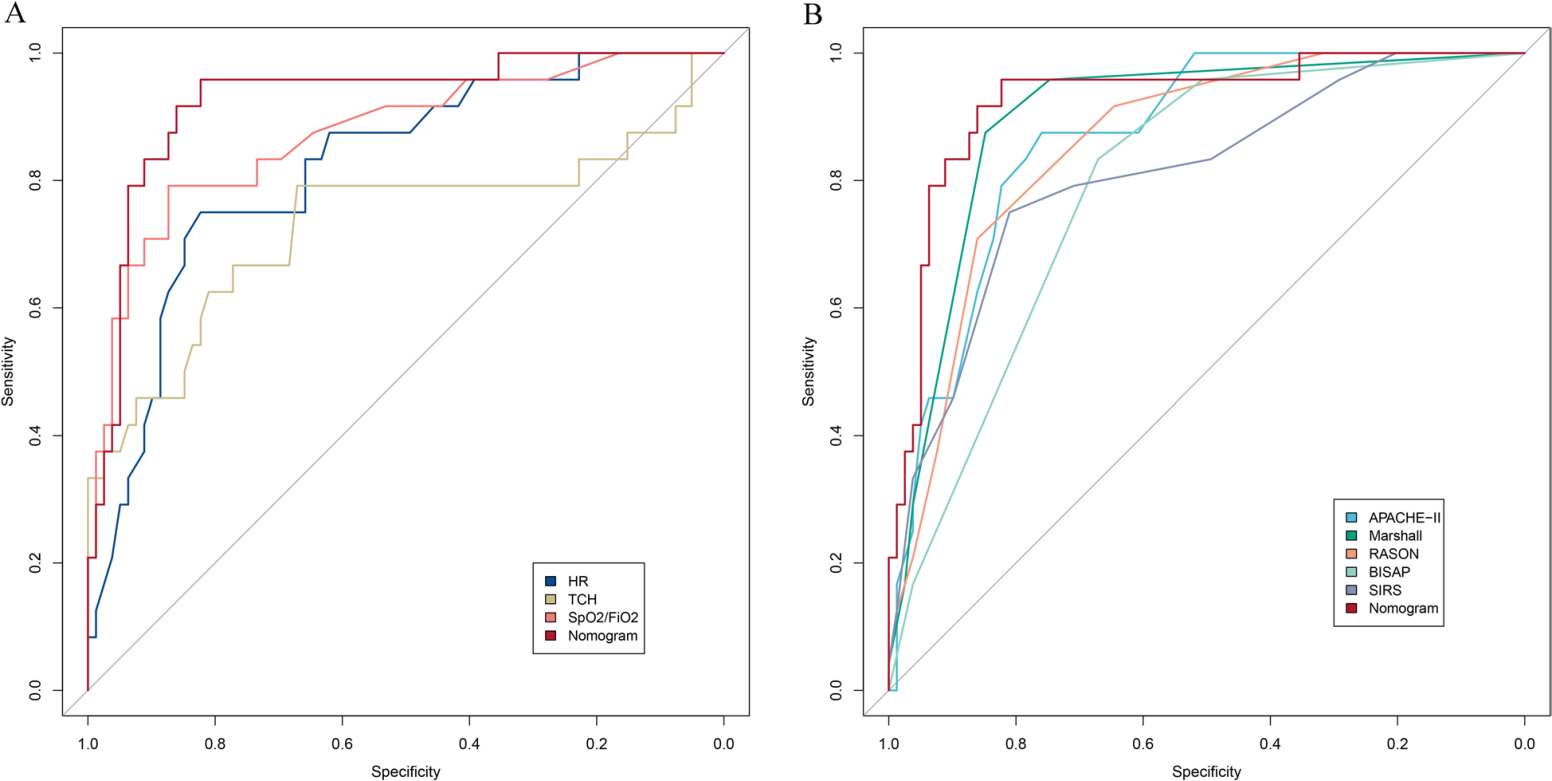
The ROC curves in prediction of ARDS related to APIP. **A** ROC curves of present prediction model; **B** ROC curves of APACHE.II, Modified Marshall, Ranson, BISAP, SIRS and Nomogram

To further confirm the role of the new model in the predictive ability of APIP-related ARDS, ROC curve analysis was performed (Table 3, Fig. 2). We found that the AUC of the new combination model was superior to that of its constituent variables (HR, TCH, SpO_2_/FiO_2_) (Fig. 2A). In addition, the new model had a strong discriminatory ability for ARDS related to APIP compared with previous scoring systems. The AUC of the new model was 0.926 (95% CI = 0.864—0.988), which was superior to that of the APACHE-II (AUC = 0.867, 95% CI 0.795—0.939), Modified Marshall (AUC = 0.896, 95% CI 0.831—0.961), Ranson (AUC = 0.857, 95%CI 0.783—0.932), BISAP (AUC = 0.792, 95%CI 0.708—0.876) and SIRS (AUC = 0.809, 95%CI 0.708—0.910)(Table 3, Fig. 2B). The Delong test results are detailed in E_Table 3. Notably, the new model performed best (sensitivity = 0.823, specificity = 0.958) when the predictive cut-off for ARDS related to APIP was set at 0.205 (Table 3).

**Table 3:** Receiver operating characteristic curves for predicting ARDS related to APIP

Characteristic	AUC	95%CI	*p* value	cutoff	Sensitivity	Specificity	Youden index
HR	0.819	0.724–0.915	< 0.001	121.500	0.823	0.750	0.573
TCH	0.730	0.588–0.873	< 0.001	8.280	0.671	0.792	0.463
SpO_2_/FiO_2_	0.873	0.785–0.960	< 0.001	289.500	0.873	0.792	0.665
APACHE.II	0.867	0.795–0.939	< 0.001	7.500	0.759	0.875	0.634
Modified Marshall	0.896	0.831–0.961	< 0.001	1.500	0.848	0.875	0.723
RASON	0.857	0.783–0.932	< 0.001	2.500	0.861	0.708	0.569
BISAP	0.792	0.708–0.876	< 0.001	1.500	0.671	0.833	0.504
SIRS	0.809	0.708–0.910	< 0.001	5.500	0.810	0.750	0.560
Nomogram	0.926	0.864–0.988	< 0.001	0.205	0.823	0.958	0.781

*p* value < 0.05 was considered statistically significant

*ARDS* acute respiratory distress syndrome, *APIP* acute pancreatitis in pregnancy, *AUC* area under the curve, *CI* confidence interval, *HR* heart rate, *TCH* Total cholesterol, *SpO*_*2*_ oxygen saturation as measured by pulse oximetry, *FiO*_*2*_ fraction of inspired oxygen, *APACHE.II* acute physiology and chronic health evaluationII, Modified Marshall, modified Marshall scoring system, *BISAP* bedside index of severity in acute pancreatitis, *SIRS* systemic inflammatory response syndrome

**Fig. 3:**
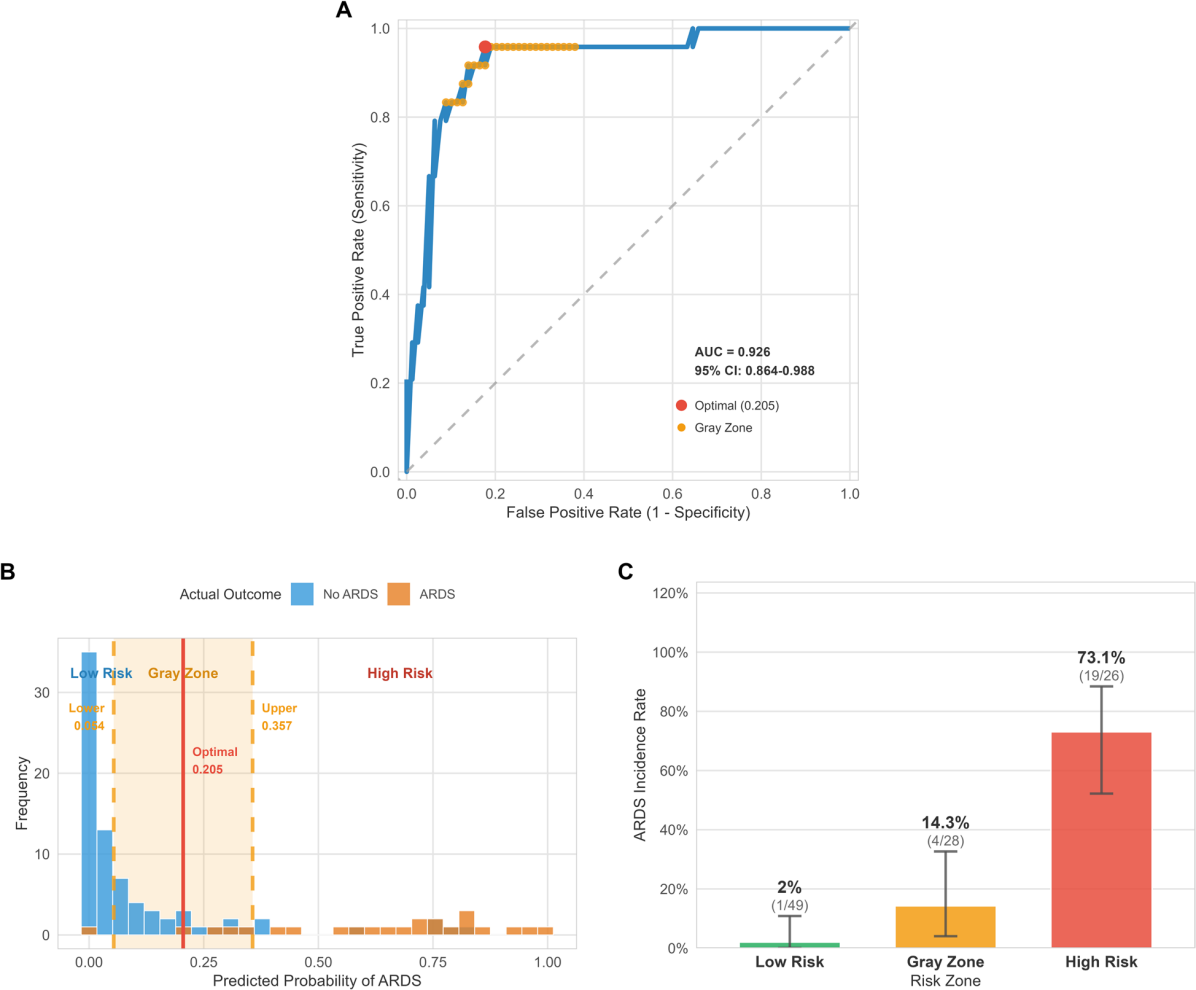
Gray zone analysis for prediction model. **A** ROC curve with gray zone annotation; **B** Predicted probability distribution with gray zone; **C** ARDS incidence rate by risk zone

To evaluate predictive accuracy and optimize clinical decision-making, a gray zone analysis based on ROC curves was conducted, classifying 103 patients into low-risk zone (47.6%, *n* = 49), gray zone (27.2%, *n* = 28), and high-risk zone (25.2%, *n* = 26). The incidence of APIP complicated with ARDS showed a significant gradient across risk zones: 2.0% (95% CI 0.1–10.9%) in low-risk zone, 14.3% (95% CI 4.0–32.7%) in gray zone, and 73.1% (95% CI 52.2–88.4%) in high-risk zone. (Fig. 3 and E_Table 4) Statistical analysis confirmed the effectiveness of stratification. The chi-square test revealed significant differences among the three groups (χ^2^ = 49.71, *P* < 0.001), and the Cochran-Armitage trend test confirmed a significant increase in ARDS incidence with rising risk levels (Z = 5.05, *P* < 0.001). (E_Table 5) For clearly classifiable patients (n = 75), the model demonstrated superior diagnostic performance: sensitivity 95.0%, specificity 87.3%, and overall accuracy 89.3%. The positive predictive value in the high-risk zone was 73.1%, while the negative predictive value in the low-risk zone reached 98.0%.(E_Table 6).

**Fig. 4:**
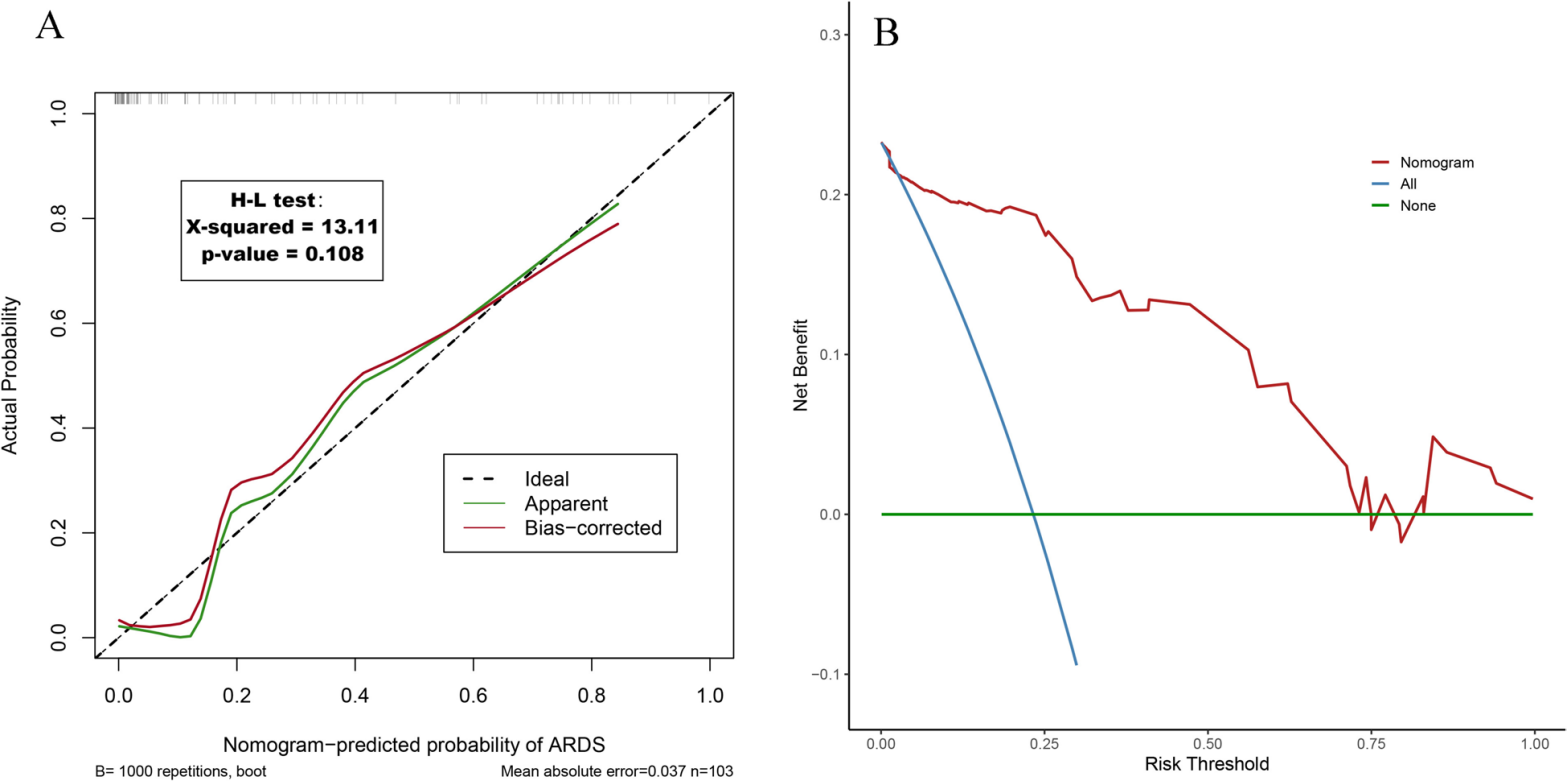
Calibration curves and decision curve analysis of new predictive models. **A** Calibration curves of acute respiratory distress syndrome (ARDS) predictive model; **B** Decision curve analysis of acute respiratory distress syndrome (ARDS) predictive model

The calibration curves showed good agreement between the nomogram estimates used to predict the risk of ARDS related to APIP and actual observations. In addition, the H–L test also showed that the difference between the predicted outcome (χ^2^ = 13.11, *p* = 0.108) and the observed outcome for APIP patients complicated with ARDS was not significant (Fig. 4A). Decision curve analysis(DCA) showed that the decision curve lies above the None and All lines when the new model’s threshold is set in the range of 2.5—70%. This indicates that there is a positive net benefit of using the new model to identify and manage patients with APIP complicated with ARDS, and the model has good clinical utility in this range(Fig. 4B).

Overall, the results of the sensitivity analysis were consistent with the preliminary analysis, which indicates that the main results were robust (E_Figure 2; E_Table 7; E_Table 8).

## Discussion

Acute pancreatitis-related ARDS during pregnancy can develop rapidly and is associated with high maternal and fetal mortality. ARDS is the earliest organ dysfunction associated with severe acute pancreatitis(SAP) [1]. Using LASSO regression and binary logistic regression analysis to identify high-risk individuals, this study indicated that a lower SpO_2_/FiO_2_ ratio, elevated HR, and elevated TCH were independent risk factors for ARDS related to APIP. A new prediction model consisting of the above three risk factors with good predictive value was built and verified. Many efforts have been made by researchers to predict the severity of AP in pregnancy [4, 15, 16], but these studies were conducted in almost single centers and did not predict the occurrence of ARDS. The present research focused on the early identification of ARDS in APIP, established a simple, practical, and accurate prediction model using routine laboratory examination and vital signs, especially the evaluation within 24 h of admission for pregnant women and fetuses.

This study identified independent risk factors associated with ARDS in APIP, highlighting a significant difference in SpO_2_/FiO_2_ ratio and heart rate (HR). The pathogenesis of acute pancreatitis (AP) and ARDS involves a cascade of acute inflammatory reactions [17, 18]. Dombernowsky et al. reported that respiratory complications are frequent in the early phase of AP, suggesting a possible association with acute lung injury due to systemic inflammation [19]. Current guidelines recommend monitoring the presence of systemic inflammatory response syndrome and vital signs at admission to predict the development of a severe disease course [20, 21]. Therefore, it is reasonable to consider HR and SpO_2_/FiO_2_ ratio as predictors of ARDS in the context of APIP. Clinicians may be able to monitor or intervene to optimize these variables to prevent or limit ARDS formation during treatment.

This study also found that the predictive ability for ARDS with a lower SpO_2_/FiO_2_ ratio was superior to that of the Ranson, APACHE II, BISAP, and SIRS scoring systems in APIP. Although arterial blood gas(ABG) measurements have been the gold standard for assessing hypoxemia in ARDS [13], it is not a routine examination in emergency departments and certain restricted areas, and some patients are unwilling to undergo invasive arterial blood collection procedures. A previous study reported that both linear and nonlinear imputations of PaO_2_:FiO_2_ from SpO_2_:FiO_2_ demonstrate good performance as long as SpO_2_ < 97% [22], and SpO_2_:FiO_2_ ratio could be considered as a diagnostic tool for ARDS for early enrollment in clinical trials [23]. Furthermore, in 2023, the global definition of ARDS was expanded to the Berlin definition, and the committee agreed to allow the use of SpO_2_:FiO_2_ as an alternative to PaO_2_:FiO_2_ for the diagnosis of ARDS [12]. To the best of our knowledge, the SpO_2_/FiO_2_ ratio as an indicator was first applied in the context of APIP in this study, predicting ARDS related to APIP in the 24-h early stage of admission, and is of great significance for the early identification of ARDS through continuous monitoring. This study not only addressed the specific context of pregnant patients, but also presented a straightforward and widely applicable predictive index of ARDS in AP that could be invaluable for clinical practice.

The present study indicated that total cholesterol level significantly increased and was an independent risk factor for ARDS related to APIP. Although Yang et al. defined a prediction model including cholesterol for moderately severe and severe AP in pregnancy [24], and a recent investigation indicated that cholesterol levels exhibit a U-shaped correlation with AP severity [25], these previous studies did not report a relationship between hypercholesterolemia and ARDS in AP. The specific mechanism by which hypercholesterolemia influences ARDS in patients with APIP remains unclear. Earlier research noted an increase in Toll-like receptor 4 (TLR4) expression in the lungs of treated mice during acute pancreatitis complicated by ARDS[26]. Hypercholesterolemia results in cholesterol buildup in macrophages and other immune cells, potentially enhancing the inflammatory response through heightened Toll-like receptor (TLR) signaling or activation of the inflammasome, leading to intensified inflammatory cascades and activation of NLRP3(NOD-, LRR-, and pyrin domain-containing 3) [27–29]. One plausible explanation is that hypercholesterolemia exacerbates ARDS associated with APIP by amplifying Toll-like receptor signaling and activating the NLRP3 inflammasome. Hypercholesterolemia is involved in the pathogenesis of ARDS and AP, indicating the potential for the development of novel drugs for treatment. Early intervention for hyperlipidemia during pregnancy could potentially mitigate the risk of ARDS in patients with APIP. Thus, the detection of serum cholesterol levels is important in the prediction of ARDS related to APIP.

This study further developed a new predictive model for ARDS related to APIP, including three variables (HR,TCH, and SpO_2_/FiO_2_). The predictive power of the new model was superior to that of the APACHE-II, Modified Marshall, Ranson, BISAP and SIRS scoring systems. In previous studies, models dealing with risk prediction of ARDS in SAP were constructed based on CT examination or AP in pregnancy [30, 31]. In a large central prospective cohort study, 28% of patients with severe pancreatitis received treatment in routine nursing units [32]. When performing diagnostic and therapeutic procedures, their side effects on the fetus should be considered, and particularly risky ones should be undertaken only when the potential benefits outweigh the risks[33]. This study reported only 54 out of 103 pregnant patients underwent chest radiography or computed tomography, including IPAP diagnosed with ARDS (24 cases).Therefore, chest radiography or CT examination may not be suitable for the early assessment of ARDS in APIP. These existing scoring systems include various complex variables and radiological examinations that have not been adjusted according to the different physiological states of pregnant women. This original model, which consisted of fewer parameters and without radiology findings, proved to be more accurate and practical for APIP. It fills this gap in the literature by providing insights tailored to the pregnant population, enhancing the ability of clinicians to identify at-risk individuals, and promoting timely intervention.

In addition, this study integrated a gray zone analysis strategy into the ARDS prediction model for APIP, effectively overcoming the limitations of traditional binary classification models and improving the model's practicality and safety. The 73.1% positive predictive value in the high-risk zone established a foundation for intervention, while the 98.0% negative predictive value in the low-risk zone ensured nursing safety. Patients within the gray zone required dynamic assessment and individualized decision-making. This high-confidence classification outcome reinforced physicians' trust in the predictive outcomes. The gray zone analysis provided tailored management plans for patients with varying risk levels, enhanced resource allocation efficiency, and offered a scientific approach to managing uncertainty.

Moreover, this study revealed that patients in the ARDS related to APIP group experienced significantly longer hospital and ICU stays, higher ventilator utilization rates, and a higher incidence of cesarean sections than patients without ARDS. Additionally, there were notable differences in fetal outcomes, with significantly elevated rates of fetal loss and premature birth. Newborns also showed significantly lower Apgar scores at 0 min. These findings underscore the importance of predictive indicators and models for ARDS in APIP, which may help emergency physicians, obstetricians, gynecologists, and ICU specialists formulate timely management strategies aimed at reducing morbidity and mortality among this vulnerable patient population.

There were several limitations to this analysis. First, it was a retrospective study with a small sample size due to the low incidence of ARDS in APIP, which may impact the robustness of our findings; the results still provide valuable scientific evidence and insights. Further prospective studies with larger sample sizes and appropriate power calculations are required to validate and refine our conclusions. Second, not all patients underwent chest imaging in their medical records to evaluate the BISAP score, and certain variables such as BUN, Scr, Hct, and albumin levels in pregnant women can be altered under specific physiological conditions [34]. Consequently, the APACHE II and Ranson scores may have low scores, potentially affecting the accuracy of assessment using these scores.Third, fluid balance, cardiac ultrasound, ventilator and Renal replacement therapy (RRT) parameters, and lung ultrasound are crucial for understanding ARDS's pathophysiological complexity. These were excluded from early predictive models due to their limited availability at admission. Future research should incorporate these multidimensional data for more accurate predictive models, aiding clinicians' decision-making.

Additionally, the study focused solely on pregnant Chinese Han women. Pulse oximetry may overestimate blood oxygen levels in individuals with darker skin compared to SaO2, and evidence for biases in other skin tones is less certain [35]. High low-density lipoprotein cholesterol is more prevalent among certain Asian groups than non-Hispanic whites [36].How racial and ethnic differences affect the accuracy of pulse oximetry and cholesterol metabolism requires further research, along with recommendations for multi-ethnic validation.

## Conclusion

This study developed a new accurate utility predictive model of ARDS related to APIP in the early phase of admission, including three simple variables (HR, TCH, and SpO_2_/FiO_2_). The key finding is that the SpO_2_/FiO_2_ ratio at admission as an independent risk factor for ARDS related to APIP should be monitored continuously and non-invasively. Furthermore, elevated serum total cholesterol was first identified as an independent risk factor for ARDS, providing a reference for the future prevention and treatment of ARDS and pancreatitis. Further prospective studies are required to optimize the model to improve the outcomes of patients with ARDS and APIP.

## Supplementary Information

Additional file 1: E-Figure 1 Identifying predictors of ARDS in patients with AP with the least absolute and selection operator regression models.Plot of the error rate of the cross-validation.Least absolute shrinkage operator coefficient profile

Additional file 2: E-Figure 2 Sensitivity analysis for the ROC curves in prediction of ARDS in APIP

Additional file 3.

## Data Availability

The data supporting the findings of this study are available from the corresponding author upon reasonable request. The data were not publicly available because of privacy and ethical restrictions.

## References

[1] Ibadov RA, Arifjanov AS, Khamdamovich S, et al. Acute respiratory distress-syndrome in the general complications of severe acute pancreatitis. Ann Hepatobiliary Pancreat Surg. 2019;23:359–64. 31825002 10.14701/ahbps.2019.23.4.359 PMC6893050

[2] Lin FY, Lu RL, Han DD, et al. A prediction model for acute respiratory distress syndrome among patients with severe acute pancreatitis: a retrospective analysis. Ther Adv Respir Dis. 2022;16:1–12. 10.1177/17534666221122592 PMC9459476 36065909

[3] Luo L, Zen H, Xu H, et al. Clinical characteristics of acute pancreatitis in pregnancy: experience based on 121 cases. Arch Gynecol Obstet. 2018;297:333–9. 29164335 10.1007/s00404-017-4558-7 PMC5778161

[4] Shi XL, Hu YP, Pu N, et al. Risk factors for fetal death and maternal AP severity in acute pancreatitis in pregnancy. Front Pediatr. 2021;9:769400. 34926347 10.3389/fped.2021.769400 PMC8674812

[5] Sinha P, Delucchi KL, McAuley DF, et al. Development and validation of parsimonious algorithms to classify acute respiratory distress syndrome phenotypes: a secondary analysis of randomised controlled trials. Lancet Respir Med. 2020;8:247–57. 31948926 10.1016/S2213-2600(19)30369-8 PMC7543720

[6] Reilly JP, Wang F, Jones TK, et al. Plasma angiopoietin-2 as a potential causal marker in sepsis-associated ARDS development: evidence from Mendelian randomization and mediation analysis. Intensive Care Med. 2018;44:1849–58. 30343317 10.1007/s00134-018-5328-0 PMC6697901

[7] Mounzer R, Langmead CJ, Wu Bu, et al. Comparison of existing clinical scoring systems to predict persistent organ failure in patients with acute pancreatitis. Gastroenterology. 2012;142:1476–82. 22425589 10.1053/j.gastro.2012.03.005

[8] Xu Z, Wu GM, Li Q, et al. Predictive value of combined LIPS and ANG-2 level in critically ill patients with ARDS risk factors. Mediators Inflamm. 2018. 10.1155/2018/1739615. 30008611 10.1155/2018/1739615 PMC6020511

[9] Anan K, Ichikado K, Ishihara T, et al. A scoring system with high-resolutioncomputed tomography to predict drug-associated acute respiratory distress syndrome: development and internal validation. Sci Rep. 2019;9:8601. 31197186 10.1038/s41598-019-45063-9 PMC6565715

[10] Zhang WY, Chang YJ, Ding YN, et al. To establish an early prediction model for acute respiratory distress syndrome in severe acute pancreatitis using machine learning algorithm. J Clin Med. 2023;12:1718. 36902504 10.3390/jcm12051718 PMC10002486

[11] Li YL, Zhang DD, Xiong YY, et al. Development and external validation of models to predict acute respiratory distress syndrome related to severe acute pancreatitis. World J Gastroenterol. 2022;28(19):2123–36. 35664037 10.3748/wjg.v28.i19.2123 PMC9134137

[12] Matthay MA, Arabi Y, Arroliga AC, et al. A new global definition of acute respiratory distress syndrome. Am J Respir Crit Care Med. 2024;209(1):37–47. 37487152 10.1164/rccm.202303-0558WS PMC10870872

[13] Ranieri VM, Rubenfeld GD, Thompson BT, ARDS Definition Task Force, et al. Acute respiratory distress syndrome: The Berlin definition. JAMA. 2012;307:2526–33. 22797452 10.1001/jama.2012.5669

[14] Banks PA, Bollen TL, Dervenis C, et al. Classification of acute pancreatitis–2012: revision of the Atlanta classification and definitions by international consensus. Gut. 2013;62:102–11. 23100216 10.1136/gutjnl-2012-302779

[15] Wang Y, Qu GB, Wu ZB, et al. Early predictive value of scoring systems and routine laboratory tests in severity and prognosis of acute pancreatitis in pregnancy. Ther Adv Gastroenterol. 2023;16:1–14. 10.1177/17562848231167277 PMC10126706 37113191

[16] Yang D, Lu H, Liu Y, et al. Development and validation of a prediction model for moderately severe and severe acute pancreatitis in pregnancy. World J Gastroenterol. 2022;28:1588–600. 35582133 10.3748/wjg.v28.i15.1588 PMC9048464

[17] Boxhoorn L, Voermans RP, Bouwense SA, et al. Acute pancreatitis. Lancet. 2020;396:726–34. 32891214 10.1016/S0140-6736(20)31310-6

[18] Thompson BT, Chambers RC, Liu KD. Acute respiratory distress syndrome. N Engl J Med. 2017;377:562–72. 28792873 10.1056/NEJMra1608077

[19] Dombernowsky T, Kristensen MO, Rysgaard S, et al. Risk factors for and impact of respiratory failure on mortality in the early phase of acute pancreatitis. Pancreatology. 2016;16:756–60. 27424478 10.1016/j.pan.2016.06.664

[20] Working Group IAP/APA acute pancreatitis guidelines. IAP/APA evidence-based guidelines for the management of acute pancreatitis. Pancreatology. 2013;13:e1-15. 24054878 10.1016/j.pan.2013.07.063

[21] Pancreatic surgery group, Chinese Society of surgery, Chinese Medical Association. The guideline for the diagnosis and treatment of severe acute pancreatitis. Chin J Surg. 2021;59(7):578–87. 34256457 10.3760/cma.j.cn112139-20210416-00172

[22] Brown SM, Grissom CK, Moss M, NIH/NHLBI PETAL network collaborators, et al. Nonlinear imputation of PaO_2_/FiO_2_ from SpO_2_/FiO_2_ among patients with acute respiratory distress syndrome. Chest. 2016;150:307–13. 10.1016/j.chest.2016.01.003 PMC4980543 26836924

[23] Chen W, Janz DR, Shaver CM, et al. Clinical characteristics and outcomes are similar in ARDS diagnosed by oxygen saturation/FiO_2_ ratio compared with PaO_2_/FiO_2_ ratio. Chest. 2015;148:1477–83. 26271028 10.1378/chest.15-0169 PMC4665739

[24] Yang DJ, Lu HM, et al. Development and validation of a prediction model for moderately severe and severe acute pancreatitis in pregnancy. World J Gastroenterol. 2022;28:1588–600. 35582133 10.3748/wjg.v28.i15.1588 PMC9048464

[25] Hong W, Zimmer V, Basharat Z, et al. Association of total cholesterol with severe acute pancreatitis: a U-shaped relationship. Clin Nutr. 2020;39:250–7. 30772093 10.1016/j.clnu.2019.01.022

[26] Matsuda N, Nishihira J, Takahashi Y, et al. Role of macrophage migration inhibitory factor in acute lung injury in mice with acute pancreatitis complicated by endotoxemia. Am J Respir Cell Mol Biol. 2006;35:198–205. 16574946 10.1165/rcmb.2005-0272OC

[27] Tall AR, Yvan-Charvet L. Cholesterol, inflammation and innate immunity. Nat Rev Immunol. 2015;15(2):104–16. 25614320 10.1038/nri3793 PMC4669071

[28] Duewell P, Kono H, Rayner KJ, et al. Nlrp3 inflammasomes are required for atherogenesis and activated by cholesterol crystals. Nature. 2010;464:1357–61. 20428172 10.1038/nature08938 PMC2946640

[29] Sheedy FJ, Grebe A, Rayner KJ, et al. CD36 coordinates NLRP3 inflammasome activation by facilitating intracellular nucleation of soluble ligands into particulate ligands in sterile inflammation. Nat Immunol. 2013;14:812–20. 23812099 10.1038/ni.2639 PMC3720827

[30] Fei Y, Gao K, Li WQ. Prediction and evaluation of the severity of acute respiratory distress syndrome following severe acute pancreatitis using an artificial neural network algorithm model. HPB. 2019;21:891–7. 30591306 10.1016/j.hpb.2018.11.009

[31] Zhang MR, Pang M. Early prediction of acute respiratory distress syndrome complicated by acute pancreatitis based on four machine learning models. Clinics (Sao Paulo). 2023;78:100215. 37196588 10.1016/j.clinsp.2023.100215 PMC10199163

[32] Sternby H, Bolado F, Canaval-Zuleta HJ, et al. Determinants of severity in acute pancreatitis: a nation-wide multicenter prospective cohort study. Ann Surg. 2019;270(2):348–55. 29672416 10.1097/SLA.0000000000002766

[33] Madro A. Pancreatitis in pregnancy—comprehensive review. Int J Environ Res Public Health. 2022;19:16179. 36498253 10.3390/ijerph192316179 PMC9737239

[34] Xie X, Kong BH, Duan T. Obstetrics and gynecology. Beijing: People’s Health Publishing House; 2018. p. 39–40.

[35] Shi C, Goodall M, Dumville J, et al. The accuracy of pulse oximetry in measuring oxygen saturation by levels of skin pig-mentation: a systematic review and meta-analysis. BMC Med. 2022;20:267. 35971142 10.1186/s12916-022-02452-8 PMC9377806

[36] Frank ATH, Zhao B, Jose PO, et al. Racial/ethnic differences in dyslipidemia patterns. Circulation. 2014;129(5):570–9. 24192801 10.1161/CIRCULATIONAHA.113.005757 PMC4212818

