# Supplementary figures and images for: An early predictive model for acute respiratory distress-syndrome related to pancreatitis in pregnancy: an 8-year multicenter analysis
Source: Eur J Med Res. 2025 Oct 7;30:931. doi: 10.1186/s40001-025-03223-w (PMC12502246; doi:10.1186/s40001-025-03223-w)

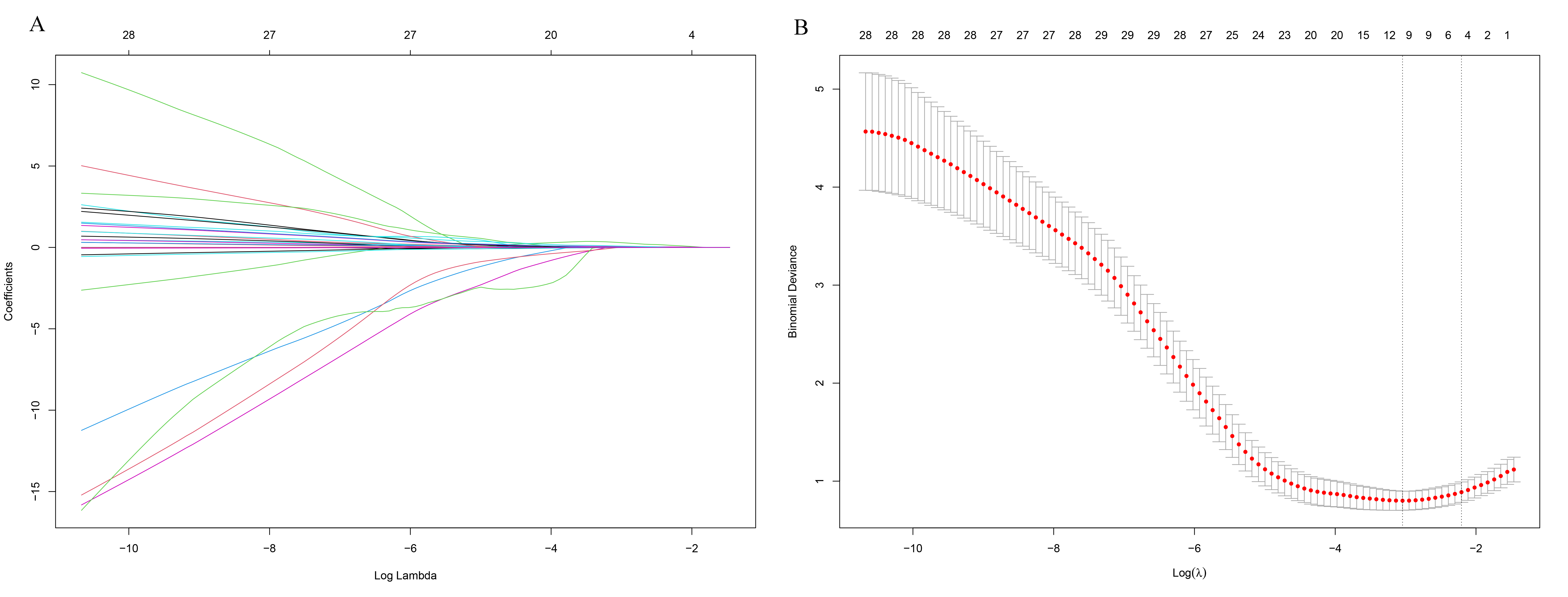

Supplement: Supplementary file 1 — Additional file 1: E-Figure 1 Identifying predictors of ARDS in patients with AP with the least absolute and selection operator regression models.Plot of the error rate of the cross-validation.Least absolute shrinkage operator coefficient profile [file 40001_2025_3223_MOESM1_ESM.tif]

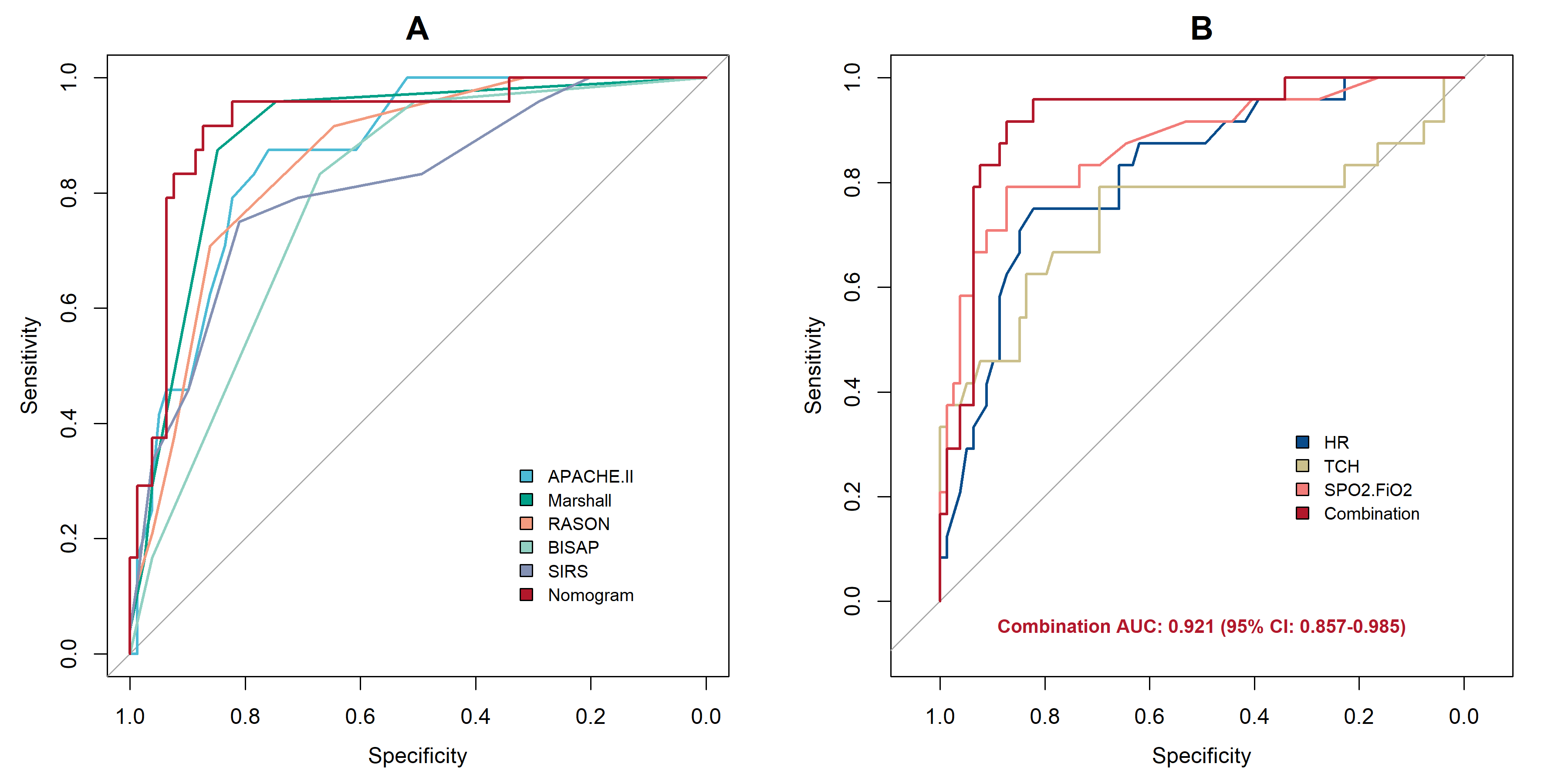

Supplement: Supplementary file 2 — Additional file 2: E-Figure 2 Sensitivity analysis for the ROC curves in prediction of ARDS in APIP [file 40001_2025_3223_MOESM2_ESM.tif]
