# Supplementary material for: An early predictive model for acute respiratory distress-syndrome related to pancreatitis in pregnancy: an 8-year multicenter analysis
Source: Eur J Med Res. 2025 Oct 7;30:931. doi: 10.1186/s40001-025-03223-w (PMC12502246; doi:10.1186/s40001-025-03223-w)
Supplement: Supplementary file 3 — Additional file 3. [file 40001_2025_3223_MOESM3_ESM.docx]

E_Table 1. LASSO regression analysis to extract predictors.

| **Characteristic** | **lambda.1se** |
| --- | --- |
| (Intercept) | -4.467719232 |
| Age | 0 |
| BMI | 0 |
| Gestation.weeks | 0 |
| Trimester.of.pregancy | 0 |
| Etiology | 0 |
| Gestational hypertension | 0 |
| Gestational diabetes | 0 |
| Gravidity | 0 |
| Time of occurrence | 0 |
| **Temperature(℃)** | 0.106892692 |
| **HR（bpm）** | 0.006606594 |
| RR（bpm） | 0 |
| MAP(mmHg) | 0 |
| WBC(*10^9) | 0 |
| NLR | 0 |
| PLR | 0 |
| Hct(%) | 0 |
| ALB(g/L) | 0 |
| TBIL(umol/L) | 0 |
| Scr(umol/L) | 0 |
| BUN(mmol/L) | 0 |
| Calcium(mmol/L) | 0 |
| CRP(mg/L) | 0 |
| PCT(ng/L) | 0 |
| **TCH(mmol/L)** | 0.026993281 |
| TG(mmol/L) | 0 |
| Glucose(mmol/L) | 0 |
| LDH(u/L) | 0 |
| PH | 0 |
| HCO_3_(mmol/L) | 0 |
| Lac (mmol/L) | 0 |
| **SpO_2_/FiO_2_** | -0.005526551 |
| LASSO, least absolute shrinkage and selection operator; BMI, body mass index; HR, heart rate; RR, respiratory rate; MAP, mean arterial pressure; NLR, neutrophil/lymphocyte ratio; PLR, platelet-to-lymphocyte ratio; Hct, hematocrit ; Scr, serum creatinine; TBIL, total bilirubin; ALB, albumin; BUN, blood urea nitrogen; CRP, C-reactive protein; PCT, procalcitonin; TCH, total cholesterol; TG, triglyceride; LDH, Lactate dehydrogenase; Lac, lactate; SpO_2_,oxygen saturation as measured by pulse oximetry; FiO_2_, fraction of inspired oxygen. | |

E_Table 2. Multicollinearity Diagnostic Statistics for LASSO-Selected Variables

| Variable | VIF | Tolerance | Collinearity Status | Interpretation |
| --- | --- | --- | --- | --- |
| Temperature (°C) | 1.88 | 0.53 | No collinearity | Low correlation with other predictors |
| SpO₂/FiO₂ ratio | 1.68 | 0.60 | No collinearity | Low correlation with other predictors |
| Heart rate (bpm) | 2.01 | 0.50 | No collinearity | Low correlation with other predictors |
| Total cholesterol (mmol/L) | 1.33 | 0.75 | No collinearity | Low correlation with other predictors |
| VIF, variance inflation factor; SpO₂/FiO₂, oxygen saturation as measured by pulse oximetry to fraction of inspired oxygen ratio. Assessment criteria: VIF < 10 and tolerance > 0.1 indicate absence of problematic multicollinearity. All variables met the criteria for inclusion in multivariable logistic regression analysis. | | | | |

E_Table 3. Delong test for comparison of AUC of different models

| **Characteristic** | **Z statistic** | ***p* value** |
| --- | --- | --- |
| Nomogram and APACHE.II | -1.212 | 0.226 |
| Nomogram and Modified Marshall | -0.653 | 0.514 |
| Nomogram and RASON | -0.420 | 0.156 |
| Nomogram and BISAP | -2.669 | 0.008 |
| Nomogram and SIRS | -2.021 | 0.043 |
| AUC, area under the curve; APACHE.Ⅱ, acute physiology and chronic health evaluationⅡ; Modified Marshall, modified Marshall scoring system; BISAP, bedside index of severity in acute pancreatitis; SIRS, systemic inflammatory response syndrome score.  *p* value < 0.05 was considered statistically significant. | | |

E_Table 4. Risk zone classification performance and clinical outcomes

| Risk Zone | APIP Patients, n (%) | ARDS Events, | Non-ARDS Events | ARDS Rate, % (95% CI) | Mean Predicted Probability |
| --- | --- | --- | --- | --- | --- |
| Low Risk | 49 (47.6) | 1 | 48 | 2.0 (0.1-10.9) | 0.013 |
| Gray Zone | 28 (27.2) | 4 | 24 | 14.3 (4.0-32.7) | 0.177 |
| High Risk | 26 (25.2) | 19 | 7 | 73.1 (52.2-88.4) | 0.709 |
| Total | 103 (100.0) | 24 | 79 | 23.3 |  |
| APIP, acute pancreatitis in pregnancy; ARDS, acute respiratory distress syndrome; CI, confidence interval. | | | | | |

E_Table 5. Statistical Comparisons Between Risk Zones

| Comparison | Test | Statistic | P value |
| --- | --- | --- | --- |
| Overall Group Comparison |  |  |  |
| ARDS rates across three zones | Chi-square test | χ² = 49.71 | <0.001 |
| Linear trend in ARDS occurrence | Cochran-Armitage trend test | Z = 5.05 | <0.001 |
| Pairwise Comparisons |  |  |  |
| Low Risk vs Gray Zone | Fisher's exact test |  | 0.056 |
| Gray Zone vs High Risk | Fisher's exact test |  | <0.001 |
| Low Risk vs High Risk | Fisher's exact test |  | <0.001 |
| ARDS, acute respiratory distress syndrome; P value < .05 was considered statistically significant. | | | |

E_Table 6. Diagnostic Performance Metrics by Risk Zone

| Risk Zone | n | Performance Metric | Value (%) | 95% CI |
| --- | --- | --- | --- | --- |
| High Risk | 26 | Positive Predictive Value | 73.1 | 52.2-88.4 |
| Low Risk | 49 | Negative Predictive Value | 98.0 | 89.1-99.9 |
| Binary Classification* | 75 | Sensitivity | 95.0 | 75.1-99.9 |
|  |  | Specificity | 87.3 | 75.5-94.7 |
|  |  | Overall Accuracy | 89.3 | 80.1-95.3 |
| *Binary classification performance calculated after excluding 28 gray zone patients (27.2% of total cohort). | | | | |

E_Table 7. Sensitivity analysis for logistic Regression Model for predicting ARDS in APIP patients.

| **Characteristic** | **Univariate** | | | **Multivariate** | | |
| --- | --- | --- | --- | --- | --- | --- |
|  | **Odds ratio** | **95%CI** | ***p* value** | **Odds ratio** | **95%CI** | ***p* value** |
| Temperature | 4.42 | 2.21-8.85 | <0.001 | 1.39 | 0.54-3.60 | 0.501 |
| HR | 1.07 | 1.03-1.10 | <0.001 | 1.04 | 1.00-1.09 | 0.050 |
| TCH | 1.15 | 1.08-1.23 | <0.001 | 1.10 | 1.01-1.19 | 0.022 |
| SPO_2_/FiO_2_ | 0.98 | 0.97-0.99 | <0.001 | 0.98 | 0.97-0.99 | 0.0045 |
| ARDS, acute respiratory distress syndrome; APIP, acute pancreatitis in pregnancy; AUC, area under the curve; CI, confidence interval; HR, heart rate; TCH, Total cholesterol; SPO_2_, oxygen saturation as measured by pulse oximetry; FiO_2_, fraction of inspired oxygen.  *p* value < 0.05 was considered statistically significant. | | | | | | |

E_Table 8. Sensitivity analysis for predictive performance of different characteristics.

| **Characteristic** | **AUC** | **95%CI** | ***P* value** | **cutoff** | **Sensitivity** | **Specificity** | **Youden index** |
| --- | --- | --- | --- | --- | --- | --- | --- |
| HR | 0.819 | (0.724-0.915) | <0.001 | 121.500 | 0.750 | 0.823 | 0.573 |
| TCH | 0.736 | (0.593-0.879) | <0.001 | 8.280 | 0.792 | 0.696 | 0.488 |
| SPO_2_/FiO_2_ | 0.873 | (0.785-0.960) | <0.001 | 289.500 | 0.792 | 0.873 | 0.665 |
| **Combination** | **0.921** | **(0.857-0.985)** | <0.001 | **0.269** | **0.917** | **0.873** | **0.790** |
| APACHE II | 0.867 | (0.795-0.939) | <0.001 | 7.500 | 0.875 | 0.759 | 0.634 |
| Marshall | 0.896 | (0.831-0.961) | <0.001 | 1.500 | 0.875 | 0.848 | 0.723 |
| RASON | 0.857 | (0.783-0.932) | <0.001 | 2.500 | 0.708 | 0.861 | 0.569 |
| BISAP | 0.792 | (0.708-0.876) | <0.001 | 1.500 | 0.833 | 0.671 | 0.504 |
| SIRS | 0.809 | (0.708-0.910) | <0.001 | 5.500 | 0.750 | 0.810 | 0.560 |
| ARDS, acute respiratory distress syndrome; APIP, acute pancreatitis in pregnancy; AUC, area under the curve; CI, confidence interval; HR, heart rate; TCH, Total cholesterol; SPO_2_, oxygen saturation as measured by pulse oximetry; FiO_2_, fraction of inspired oxygen. APACHE.Ⅱ, acute physiology and chronic health evaluationⅡ; Marshall, modified Marshall scoring system; BISAP, bedside index of severity in acute pancreatitis; SIRS, systemic inflammatory response syndrome score.  *p* value < .05 was considered statistically significant. | | | | | | | |
